# Developmental Origin of Oligodendrocyte Lineage Cells Determines Response to Demyelination and Susceptibility to Age-Associated Functional Decline

**DOI:** 10.1016/j.celrep.2016.03.069

**Published:** 2016-04-14

**Authors:** Abbe H. Crawford, Richa B. Tripathi, William D. Richardson, Robin J.M. Franklin

**Affiliations:** 1 Wellcome Trust-MRC Cambridge Stem Cell Institute and Department of Clinical Neurosciences, Clifford Allbutt Building, Cambridge Biomedical Campus, University of Cambridge, Cambridge CB2 0AH, UK; 2 Wolfson Institute for Biomedical Research, University College London (UCL), Gower Street, London WC1E 6BT, UK

**Keywords:** myelin, oligodendrocyte, glial diversity, remyelination, oligodendrocyte progenitor

## Abstract

Oligodendrocyte progenitors (OPs) arise from distinct ventral and dorsal domains within the ventricular germinal zones of the embryonic CNS. The functional significance, if any, of these different populations is not known. Using dual-color reporter mice to distinguish ventrally and dorsally derived OPs, we show that, in response to focal demyelination of the young adult spinal cord or corpus callosum, dorsally derived OPs undergo enhanced proliferation, recruitment, and differentiation as compared with their ventral counterparts, making a proportionally larger contribution to remyelination. However, with increasing age (up to 13 months), the dorsally derived OPs become less able to differentiate into mature oligodendrocytes. Comparison of dorsally and ventrally derived OPs in culture revealed inherent differences in their migration and differentiation capacities. Therefore, the responsiveness of OPs to demyelination, their contribution to remyelination, and their susceptibility to age-associated functional decline are markedly dependent on their developmental site of origin in the developing neural tube.

## Highlights

• Oligodendrocyte progenitors arise from distinct ventral and dorsal domains
• Dorsal progenitors exhibit enhanced recruitment and differentiation in remyelination
• Dorsal progenitors become less able to differentiate with aging

## Graphical Abstract

**Figure:**
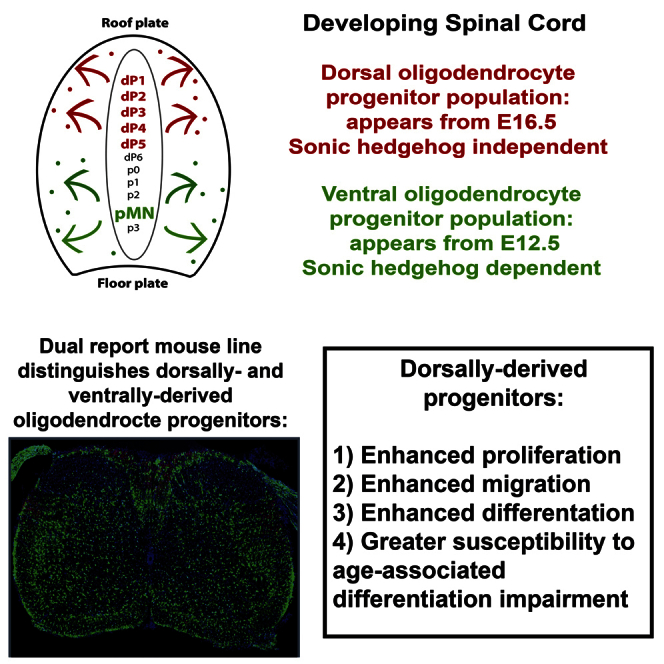

## Teaser

Oligodendrocyte progenitors (OPs) arise from distinct ventral and dorsal domains within the embryonic CNS. Crawford et al. use dual-color reporter mice to show that, in response to demyelination, dorsal OPs undergo enhanced recruitment and differentiation during remyelination. With increasing age, dorsal OPs become less able to differentiate into mature oligodendrocytes.

## Introduction

Enhancing the regeneration of myelin sheaths in the CNS is an important therapeutic goal in the management of demyelinating disease (Franklin and Gallo, 2014). Myelin sheaths facilitate efficient neurotransmission and provide trophic support to the axon, crucial to the maintenance of axonal integrity (Nave and Werner, 2014). Loss of myelin results in conduction block in the short term and axonal degeneration in the longer term (Franklin et al., 2012). Myelin regeneration, or remyelination, is mediated by oligodendrocyte progenitors (OPs) distributed throughout the CNS. OPs become activated in response to demyelination and are recruited to the damaged area where they differentiate into oligodendrocytes (OLs) (Levine and Reynolds, 1999, Tripathi et al., 2010, Zawadzka et al., 2010, Moyon et al., 2015). The newly differentiated OLs establish contact with exposed axons and ultimately generate new myelin sheaths. This process can be highly efficient, enabling complete reconstruction of the original tissue architecture and full functional recovery. However, aging is associated with a declining rate of remyelination and, in chronic disease, remyelination frequently fails due to insufficient OP differentiation (reviewed by Franklin et al., 2012). This failure of remyelination is a major contributor to disease progression and represents a significant barrier to the effective treatment of chronic demyelinating diseases. The development of effective remyelination-enhancing therapeutic strategies necessitates a detailed understanding of the biology of OPs and the mechanisms of their differentiation block in aging and disease (Huang et al., 2011).

Despite the widespread distribution of OPs throughout the CNS, the founder cells of the lineage are spatially localized in the developing neural tube and emerge as regionally and temporally discrete populations (reviewed by Richardson et al., 2006, Bergles and Richardson, 2015). In the developing mouse spinal cord, OPs initially arise around embryonic day 12.5 (E12.5) from the ventral ventricular zone (VZ), under the control of Sonic Hedgehog (Shh) from the notochord and floor plate (Pringle and Richardson, 1993, Pringle et al., 1996). From ∼E15.5, a second wave of OPs emerges from the dorsal VZ, independent of Shh signaling (Cai et al., 2005, Fogarty et al., 2005, Vallstedt et al., 2005), contributing ∼20% of the total OP population (Tripathi et al., 2011). Most dorsally derived OPs remain in the dorsal half of the cord, while the ventrally derived OPs spread widely throughout the cross-section of the cord (Tripathi et al., 2011, Zhu et al., 2011). As a result, the dorsal and dorso-lateral funiculi are initially populated by a mixture of dorsally and ventrally derived OL lineage cells. Subsequently, ventrally derived cells are displaced, through an unknown mechanism, by their dorsally derived counterparts so that the corticospinal and rubrospinal tracts, for example, become myelinated mainly by dorsally derived OLs (Tripathi et al., 2011).

In the developing forebrain, spatially distinct sources of OPs have also been identified. The first OPs appear around E12.5, emerging from *Nkx2.1*-expressing progenitor cells in the medial ganglionic eminence (MGE) and anterior entopeduncular area in the ventral telencephalon (forerunner of the forebrain). Subsequently, from around E15.5, OPs emerge from *Gsh2*-expressing progenitors of the lateral and caudal ganglionic eminences (LGE and CGE). Finally, from around the time of birth (postnatal day zero, P0), OPs start to be generated by *Emx1*-expressing progenitors in the cortical VZ. The initial wave of *Nkx2.1*-domain derived OPs is largely eliminated from the postnatal cortex, implying competition among the different populations or an ongoing turnover with preferential replacement of earlier-formed cells by their later-forming counterparts (Kessaris et al., 2006).

OLs with different spatial origins, possibly specified via different transcriptional regulators, might have distinct properties and functions in the mature brain. To investigate this possibility, we have generated a dual-reporter mouse line *Sox10-lox-eGFP-STOP-lox-TdTomato* (referred to as *Sox10-GFP-TdTom*) that allows OL lineage cells derived from different VZ domains to be distinguished by color (Tripathi et al., 2011). In the absence of Cre recombinase, all OPs and their OL progeny (*Sox10*-expressing) express GFP by default; exposure to Cre triggers recombination and activates tandem-duplicated Tomato (TdTom) expression instead. For example, crossing to *Msx3-Cre* mice (Fogarty et al., 2005) induces TdTom expression only in those OL lineage cells that are derived from the dorsal spinal cord and brainstem (Tripathi et al., 2011), while *Emx1-Cre* (Kessaris et al., 2006) induces TdTom only in OL lineage cells that originate within the developing cerebral cortex (Tripathi et al., 2011).

Both in the forebrain and spinal cord there is competition between dorsally and ventrally derived OL lineage cells. In the spinal cord, dorsally derived cells displace their ventrally derived relatives from dorsal axon tracts during postnatal life (Tripathi et al., 2011). In the forebrain, OL lineage cells derived from the MGE (*Nkx2.1*-expressing) migrate into the cortex before birth, but are largely eliminated after birth and superseded by cells from more dorsal territories (*Gsh2*- and *Emx1*-expressing; Kessaris et al., 2006). Prompted by the study by Zhu et al. (2011), which drew attention to the remyelinating capacity of dorsally derived cells, we asked whether this inter-regional competition is also evident during remyelination. We induced acute focal demyelination in the ventral funiculus of the spinal cord or in the corpus callosum by targeted lysolecithin injection and followed remyelination of the lesions using the two-color reporter system described above. We found that dorsally derived OL lineage cells dominate the remyelination response in both ventral spinal cord and corpus callosum. However, the dorsally derived cells showed an accelerated age-related impairment in their ability to differentiate into myelinating OLs compared to their ventrally derived counterparts. When cultured in vitro, the two populations demonstrated inherent differences in their migration and differentiation capacities and these functional differences were greater in adult brain-derived cells compared to cells from neonatal brain. Thus, we provide evidence that the developmental origin of OL lineage cells influences their regenerative properties in adulthood.

## Results

### Dorsally Derived OL Lineage Cells Dominate Remyelination in the Young Adult Spinal Cord

2-month-old (P58–P74, mean age P68) mice carrying the *Msx3-Cre* and *Sox10-GFP-TdTom* transgenes were used for spinal cord experiments. In *Msx3-Cre: Sox10-GFP-TdTom* spinal cords *Sox10*-expressing OPs and OLs derived from the ventral VZ are labeled constitutively with enhanced GFP, while dorsally derived OPs/OLs are labeled with TdTom (Tripathi et al., 2011). Immunolabeling spinal cord sections for the OL lineage marker Olig2 revealed that 94.7 ± 1.6% of all reporter-positive cells (either TdTom+ or GFP+) were also Olig2+, and that 96 ± 1.8% of Olig2+ cells were either TdTom+ or GFP+, confirming specific labeling of OL lineage cells in the adult CNS (Tripathi et al., 2011 and data not shown).

To determine the percentage of TdTom+ and GFP+ cells remaining as OPs in the non-lesioned spinal cord, immunofluorescent staining was performed for NG2, a surface proteoglycan found on OPs, 14.3 ± 3.9% of TdTom+ cells co-labeled with NG2, compared to 2.8 ± 0.9% of GFP+ cells (p = 0.005; Student’s t test). To determine the percentage of cells that differentiated into mature OLs, labeling for CC1 was performed, 41.3 ± 0.8% of TdTom+ cells co-labeled with CC1+, compared with 91.3 ± 1.1% (p = 0.003; Student’s t test).

**Figure 1:**
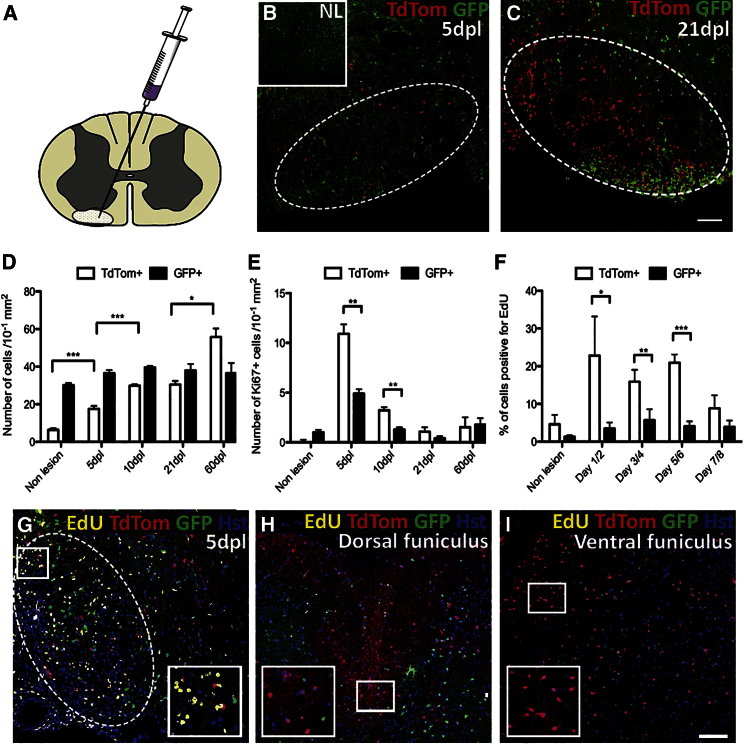
Increased Recruitment of dOPs versus vOPs into a Demyelinated Lesion in the Ventral Funiculus of the Spinal Cord (A) Schematic depiction of the location of lysolecithin injection into the ventral funiculus of the caudal thoracic spinal cord. (B and C) Immunolabeling of the non-lesioned (B, inset) and lesioned ventral funiculus of the caudal thoracic spinal cord at 5 dpl (B) and 21 dpl (C). The TdTom+ dOPs are infrequent in the non-lesioned ventral funiculus, but increase in number within the lesion (marked by the white dashed line). (D) Increase in TdTom+ dOL lineage cells following demyelination in the ventral funiculus (p < 0.001, 5–10 dpl; p = 0.023, 21–60 dpl; and one-way ANOVA and Tamhane post hoc test). There is no equivalent change in the number of GFP+ vOL lineage cells (p = 0.76). (E) There are significantly more TdTom+/Ki67+ cells than GFP+/Ki67+ cells at 5 dpl (p = 0.006 and Student’s t test) and 10 dpl (p = 0.001). (F) A significantly greater proportion of TdTom+ cells were also EdU+ on days 1/2 (p = 0.017 and one-way ANOVA and Tamhane post hoc test), days 3/4 (p = 0.003), and days 5/6 (p < 0.001), compared to GFP+ cells. (G–I) EdU was administered on days 3/4 and the animal sacrificed at 5 dpl. Abundant EdU+ cells are seen within the lesion and the immediately surrounding area (G). Almost no EdU+/TdTom+ or GFP+ cells were detected in the dorsal funiculus (H) or lateral funiculus (I). The data are presented as mean ± SEM (n = 3 mice). The scale bars represent 100 μm (^∗^p < 0.05, ^∗∗^p < 0.01, and ^∗∗∗^p < 0.001).

Demyelination was induced by the injection of lysolecithin into the ventral funiculus of the spinal cord. This induced a small focal area of demyelination which undergoes a characteristic pattern of remyelination: OP recruitment peaks around 5 days post-lesion (dpl), differentiation into myelin sheath-forming OLs commences around 10 dpl, and continues until remyelination is complete in young adult mice at around 21 dpl (Arnett et al., 2004). In the non-lesioned ventral funiculus, there were only 63 ± 8 TdTom+ cells/mm^2^ compared to 302 ± 11 GFP+ cells/mm^2^. Following induction of demyelination in the ventral funiculus, the number of TdTom+ cells increased in the lesion periphery at 5 dpl, appeared in the lesion core by 10 dpl, and were abundant throughout the lesioned area by 21 dpl (Figures 1A–1C). Cell counts confirmed that the number of TdTom+ cells within the lesion changed significantly over time, yet there was no significant change in the number of GFP+ cells (Figure 1D). By 60 dpl, the TdTom+ cells had increased in number ∼9-fold and had become the dominant population of OL lineage cells within the lesioned area (557 ± 45 TdTom+ cells/mm^2^ compared to 364 ± 55 GFP+ cells/mm^2^).

### Dorsally Derived OPs Proliferate in Response to Focal Demyelination

To assess cell proliferation in lesions and the surrounding tissue, we immunolabeled for the nuclear antigen Ki67, which is expressed through all phases of the cell cycle except early G1/G0. We found that TdTom+/Ki67+ cells were more numerous than GFP+/Ki67+ cells at both 5 dpl and 10 dpl, after which, numbers of Ki67+ cells declined to baseline levels (Figure 1E). This indicates that a greater fraction of dorsally derived OPs (dOPs) was mitotically active compared to their ventrally derived counterparts (vOPs).

To determine whether vOPs undergo an early or late proliferative response that we failed to detect at 5 dpl or 10 dpl, we performed a time course of EdU incorporation. Following lysolecithin injection into the spinal cord (ventral funiculus), mice were given EdU by intraperitoneal injection on 2 consecutive days starting at 1, 3, 5, or 7 dpl and analyzed 24 hr after the second EdU dose (on 3, 5, 7, or 9 dpl). From 3 dpl to 7 dpl, proportionally more TdTom+ dOPs than GFP+ vOPs incorporated EdU (Figure 1F). EdU+ cells were observed within the demyelinated lesion and the immediately surrounding area (Figure 1G). Very few EdU+ cells were detected in the contralateral ventral funiculus, dorsal funiculus, or lateral funiculi (Figures 1H and 1I), indicating that the prominent recruitment of dOPs into the lesioned ventral funiculus is the result of a local proliferative response.

### dOPs Undergo Enhanced OL Differentiation into Remyelinating OLs and Schwann Cells in Spinal Cord Lesions Relative to vOPs

**Figure 2:**
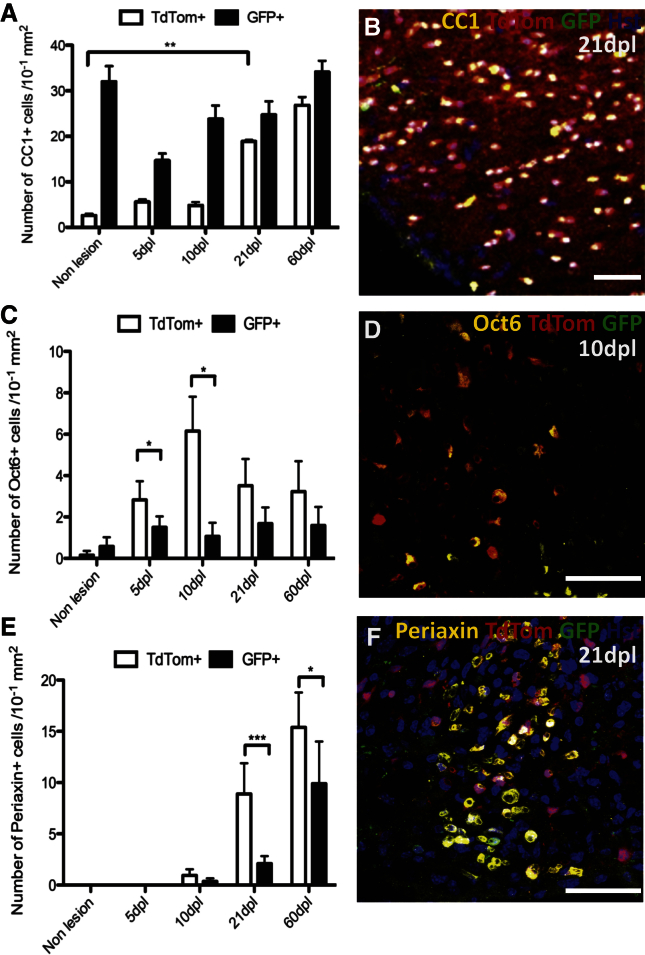
OLs and Schwann Cell Generation by dOPs and vOPs during Remyelination of Spinal Cord Demyelination (A) There is a significant increase in TdTom+, CC1+ cells between non-lesioned and lesioned tissue at 21 dpl (p = 0.001 and one-way ANOVA and Tamhane), whereas GFP+, CC1+ cells do not change in number significantly. (B) Numerous TdTom+/CC1+ cells are present within the remyelinated area. (C) There are significantly more TdTom+/Oct6+ cells than GFP+/Oct6+ cells in lesions at 5 dpl (p = 0.042 and Student’s t test) and 10 dpl (p = 0.036). (D) Oct6+/TdTom+ cells within the lesioned area. (E) There are significantly more TdTom+/Periaxin+ cells than GFP+/Periaxin+ cells in lesions at 21 dpl (p < 0.001 and Student’s t test) and 60 dpl (p = 0.045). (F) A cluster of Periaxin+ Schwann cells within the core of the remyelinated area, the majority of which co-label for TdTom. The data are presented as mean ± SEM (n = 3 mice). The scale bars represent 50 μm.

We compared the differentiation capacities of dOPs and vOPs by immunolabeling for the CC1 antigen, which is expressed by mature OLs. The number of TdTom+, CC1+ dOLs increased greatly between 5 dpl and 60 dpl, so that the density of dOLs became >10-fold higher than it had been pre-lesion (Figures 2A and 2B). In contrast, the density of GFP+, CC1+ vOLs initially decreased, but recovered to approximately the pre-lesion level (Figure 2A). Thus, the enhanced proliferation of dOPs relative to vOPs is mirrored by an increased rate of differentiation of dOPs into CC1+ OLs in the lesion.

Schwann cells contribute to CNS remyelination following lysolecithin-induced demyelination, and the majority of remyelinating Schwann cells are generated by centrally derived OPs (Zawadzka et al., 2010). To determine the relative contributions of dOPs and vOPs to Schwann cell differentiation, the lesioned tissue was immunolabeled for Oct6/SCIP, a transcription factor expressed by pre-myelinating Schwann cells, and Periaxin, a protein of myelinating Schwann cells (Sim et al., 2002a). Of the Oct6+ cells present within the lesioned area at 5 dpl or 10 dpl, more were TdTom+ than GFP+ (Figures 2C and 2D) and more Periaxin+ cells were TdTom+ than GFP+ at 21 dpl and 60 dpl (Figures 2E and 2F). For example, on 60 dpl, 37% ± 4% of TdTom+ cells expressed Periaxin, compared with 22% ± 3% of GFP+ cells, while 58% ± 4% of Periaxin+ cells were TdTom+ and 42% ± 4% were GFP+. Therefore, more remyelinating Schwann cells are generated by TdTom+ dOPs than by GFP+ vOPs.

### Dorsally Derived OLs Dominate Remyelination of the Young Adult Corpus Callosum

In the developing forebrain, OPs have been shown to emerge from the VZ in a ventral-to-dorsal wave from distinct VZ territories marked by expression of transcription factors Nkx2.1, Gsh2, and Emx1 (Kessaris et al., 2006). Nkx2.1 and Gsh2 are expressed in the MGE and LGE of the ventral forebrain, respectively, while Emx1 is expressed in the cortical VZ (dorsal forebrain). To study the relative properties of OPs derived from the ventral forebrain and the cortex, the *Sox10-GFP-TdTom* reporter was crossed onto the *Emx1-Cre* background. In double-transgenic offspring, Emx1+ dOPs (and their dOL derivatives) express TdTom, while vOPs and vOLs from the MGE and LGE constitutively express GFP. We found that 88% ± 10% of reporter-positive cells (either TdTom+ or GFP+) in the adult corpus callosum co-labeled for Olig2, and 100% ± 1% of Olig2+ cells expressed either TdTom or GFP (data not shown), confirming specific labeling of OL lineage cells.

**Figure 3:**
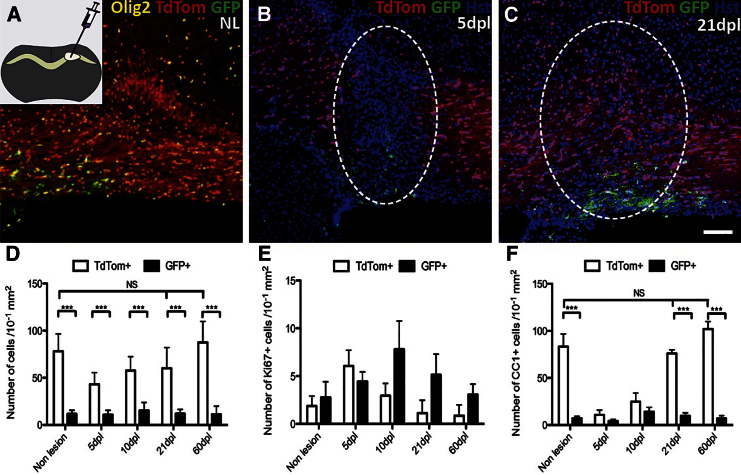
dOPs Dominate Remyelination of the Corpus Callosum (A) The non-lesioned corpus callosum is dominated by TdTom+ dorsally derived OL lineage cells, with infrequent GFP+ ventrally derived cells typically clustered over the lateral walls of the lateral ventricles. The inset shows a schematic depiction of the location of the lysolecithin injection into the corpus callosum. (B) Corpus callosum 5 days after lysolecithin injection: cellular infiltration is evident by the abundance of Hst+ nuclei. (C) Corpus callosum 21 days after lysolecithin injection: the lesioned area is fully remyelinated with a predominance of TdTom+ cells (lesioned area marked by white dashed line). (D) TdTom+ cells are more abundant than GFP+ cells within both the non-lesioned and lesioned corpus callosum (p < 0.001 at all time points and Student’s t test). The number of TdTom+ cells changed significantly over time (p < 0.001 and one-way ANOVA), while the number of GFP+ cells did not. (E) Ki67+ cells in both TdTom+ and GFP+ cell populations show a significant change in with time (p < 0.001 TdTom+, p = 0.04 GFP+, and Kruskal-Wallis test). There are no significant differences between the numbers of TdTom+ and GFP+ cells at any time point examined. (F) There are significantly more TdTom+, CC1+ cells in both the NL and lesioned corpus callosum, compared with GFP+, CC1+ cells (p < 0.001 and Student’s t test). The data are presented as mean ± SEM (n = 3 mice). The scale bars represent 100 μm.

Focal demyelination was induced by lysolecithin injection into the corpus callosum of 2-month-old mice (P64–P84, mean age P75) and the ensuing remyelination, which undergoes a similar timeline of remyelination to spinal cord demyelination (Miron et al., 2013), was analyzed as described above for spinal cord. TdTom+ (cortex-derived) dOPs and dOLs were significantly more numerous than GFP+ vOPs and vOLs within the normal corpus callosum (782 ± 185 cells/mm^2^ versus 117 ± 37 GFP+ cells/mm^2^, respectively) (Figures 3A and 3D). Following lysolecithin injection, TdTom+ cells were initially depleted (5 dpl), but their numbers increased subsequently, recovering to non-lesioned control cell densities by 21 dpl (Figures 3B–3D). GFP+ cells, in contrast, did not much change during demyelination/remyelination (Figure 3D).

Within the lesioned area of corpus callosum, the number of proliferating Ki67+ cells, both TdTom+ and GFP+, changed over time, first increasing then decreasing to pre-lesion levels (Figure 3E). The proliferative response of TdTom+ dOPs was more rapid than GFP+ vOPs, but their overall responses were similar (Figure 3E).

TdTom+, CC1+ dOLs were initially depleted (at 5 dpl), but recovered to their pre-lesion density by 21 dpl (Figure 3F). Both before lesioning and after recovery at 21 dpl and 60 dpl, TdTom+, CC1+ dOLs greatly outnumbered their ventrally derived counterparts, effectively dominating the remyelination response (Figure 3F). Unlike spinal cord lesions, Oct6+ or Periaxin+ Schwann cells were not detected in remyelinating corpus callosum lesions.

### dOPs Outperform vOPs in their Response to Demyelination in the Aging Spinal Cord

The efficiency of remyelination declines with age, and this has been associated with a reduction in the remyelinating capacity of OPs (Shields et al., 1999, Sim et al., 2002b, Shen et al., 2008). To investigate whether the developmental origin of OPs influences age-associated functional decline, focal demyelination was induced in the spinal cord (ventral funiculus) of 6-month-old mice (P176–P186, mean age P184) and 13-month-old mice (P388–P419, mean age P401). Mice were sacrificed at 5 dpl, 10 dpl, or 21 dpl. In 13-month-old mice, 92% ± 2% of Olig2+ cells were either TdTom+ or GFP+, confirming that the transgene continued to be expressed efficiently in the older spinal cord.

**Figure 4:**
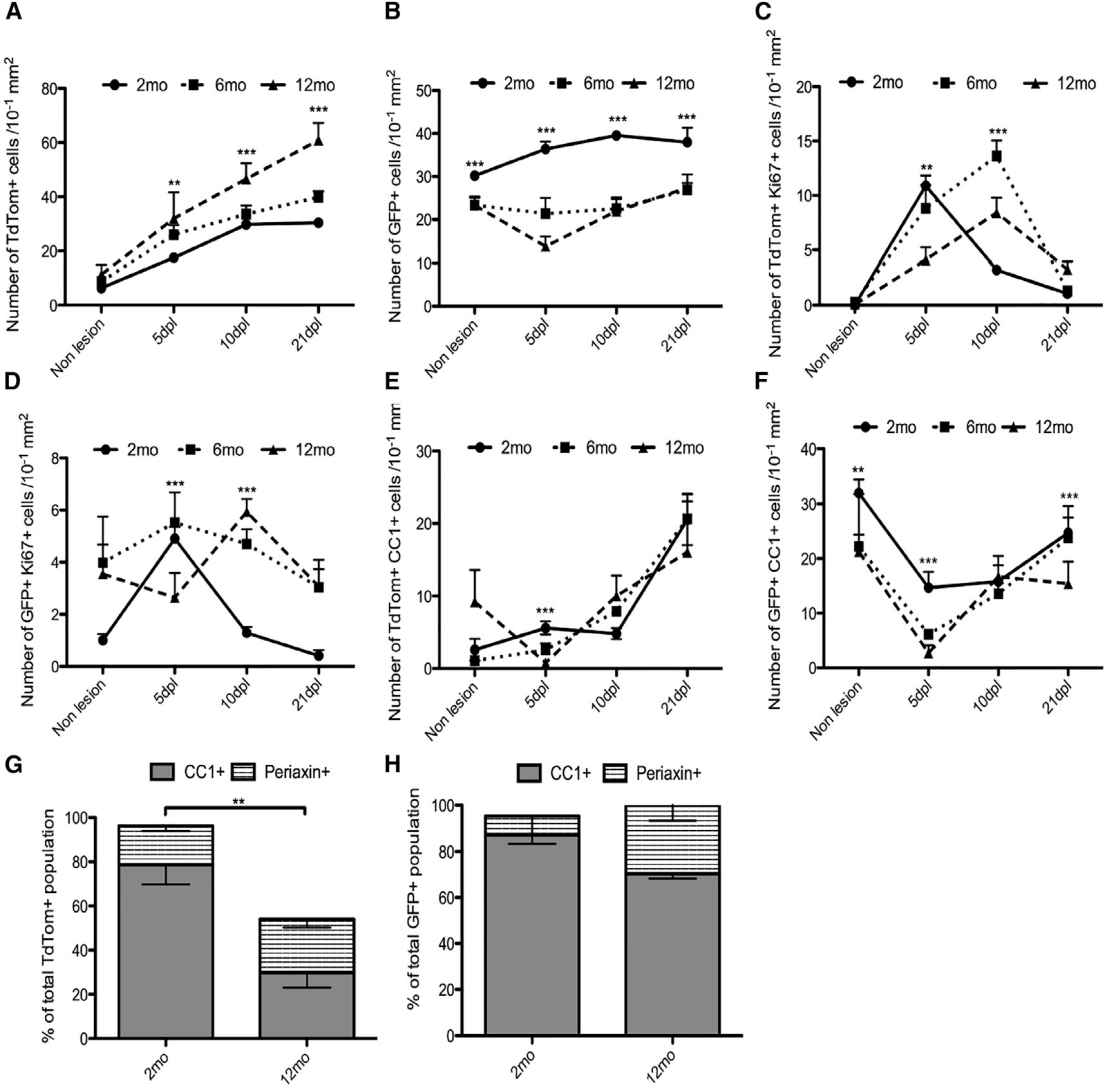
Kinetics of Remyelination in the Aging Spinal Cord (A) At all ages examined (2-, 6-, and 13-month-old mice), numbers of TdTom+ (dOL lineage) cells increase 5- to 6-fold postlesion, reaching a plateau at 10 dpl (2-month-old mice) or after 21 dpl (13-month-old mice). (B–D) In contrast, numbers of GFP+ (vOL lineage) cells do not change dramatically postlesion, except for a decrease at 5 dpl in 13-month-old mice (B). Both dOPs (C) and vOPs (D) undergo a transient proliferative response to demyelination, marked by increases in the numbers of Ki67+ OL lineage cells. The peak of proliferation is delayed in 6-month- and 13-month-old mice relative to 2-month-old mice, indicating a slower regenerative response in older animals. (E) TdTom+, CC1+ dOLs increase in number from 5 dpl to 21 dpl postlesion at all ages. The final numbers of CC1+ cells at 21 dpl are similar (p = 0.79 and one-way ANOVA). (F) Numbers of GFP+, CC1+ vOLs decrease sharply postlesion, but recover between 5 dpl and 21 dpl. (G) The fraction of dOL lineage cells that are CC1+ dOLs at 21 dpl decreases markedly with age (from ∼80% to ∼30%; p = 0.008; and Student’s t test), indicating that the rate of differentiation of dOPs is slower in older mice. (H) The fraction of vOL lineage cells that are CC1+ vOLs at 21 dpl is ∼90% in 2-month-old mice compared to ∼70% in 13-month-old mice, implying a more similar rate of differentiation of vOPs than dOPs in old versus young mice. The data are presented as mean ± SEM (n = 3 mice).

The number of TdTom+ dOL lineage cells in the non-lesioned ventral funiculus increased modestly (∼2-fold) between 2 months and 13 months (Figure 4A). Following demyelination at 2 months, 6 months, or 13 months, the number of dOL lineage cells first decreased then increased markedly from pre-lesion levels, reaching a plateau in 2-month-old animals at 10 dpl, but continuing to increase in 6-month- and 13-month-old animals until at least 21 dpl (Figure 4A). In contrast, the number of GFP+ vOL lineage cells in non-lesioned white matter was slightly less at 6 months and 13 months than at 2 months and changed only modestly or not at all following lesion induction (Figure 4B). The net result was that dOL lineage cells, which were the minority of OL lineage cells in pre-lesion white matter, came to outnumber vOL lineage cells by 21 dpl. For example, in 2-month-old mice, dOL lineage cells increased from 17% ± 2% of all OL lineage cells in pre-lesion white matter to 45% ± 3% of the total at 21 dpl; in 13-month-old mice, they increased from 33% ± 6% to 83% ± 4% over the same period. The latter might be an underestimate because the number of dOL lineage cells in 13-month-old mice was still increasing at 21 dpl (Figure 4A).

Following lesion induction, the numbers of Ki67+ dOPs and vOPs increased transiently at 5 dpl and/or 10 dpl, but declined to pre-lesion levels by 21 dpl (Figures 4C and 4D). The peak proliferative response was delayed in the 6-month- and 13-month-old mice compared to the 2-month-old mice (Figures 4C and 4D). The extent of the peak proliferative response was greater for dOPs than vOPs at all ages (Figures 4C and 4D). Therefore, both the proliferative response and the ultimate contribution to the total population was greater for dOL lineage cells than their vOL counterparts, suggesting that dOPs are more adapted for regeneration in the spinal cord.

### dOPs Differentiate Less Efficiently in Spinal Cord Lesions of Old versus Young Mice

To estimate the fraction of dOL and vOL lineage cells that differentiated into OLs in ventral funiculus lesions, we immunolabeled with antibody CC1. In 13-month-old mice, the number of TdTom+, CC1+ dOLs was decreased at 5 dpl, whereas in 2-month- and 6-month-old mice, they increased slightly (Figure 4E). In mice of all ages, dOL numbers increased between 5 dpl and 21 dpl, and the final numbers of dOLs in lesions were similar at all ages (Figure 4E). However, at 21 dpl the fraction of the total TdTom+ cell population that immunolabeled for CC1 was significantly less in 13-month-old mice than in 2-month-old mice (30% ± 7% versus 79% ± 9%) (Figure 4G). This demonstrates that in 13-month-old mice, the majority of dOL lineage cells in lesions at 21 dpl are undifferentiated dOPs and suggests that the dOPs differentiate more slowly into dOLs in old versus young animals. The fraction of the total TdTom+ population that labeled for Periaxin did not change significantly with age (18% ± 2% at 2 months versus 24% ± 4% at 13 months; p = 0.21 and Student’s t test), suggesting that Schwann cell differentiation is not affected by aging (Figure 4G).

In mice of all ages, the number of GFP+, CC1+ vOLs decreased during the first 5 dpl, then increased between 5 dpl and 21 dpl (Figure 4F). At 21 dpl, the fractions of all vOL lineage cells that were CC1+ showed a slight decrease with age (87 ± 4% at 2 months and 70 ± 2% at 13 months) (Figure 4H). However, the proportion of GFP+ cells that labeled for Periaxin showed a significant increase in the 13-month-old mice (8 ± 10%) compared with 2-month-old mice (30 ± 7%) (Figure 4H). Thus, it appears that in contrast to dOPs, vOPs are recruited less efficiently to spinal cord lesions in older mice, but their rate of differentiation is not significantly compromised by age.

### Dorsal OPs Show a Declining Efficiency of Recruitment and Differentiation following Focal Demyelination of the Aging Corpus Callosum

**Figure 5:**
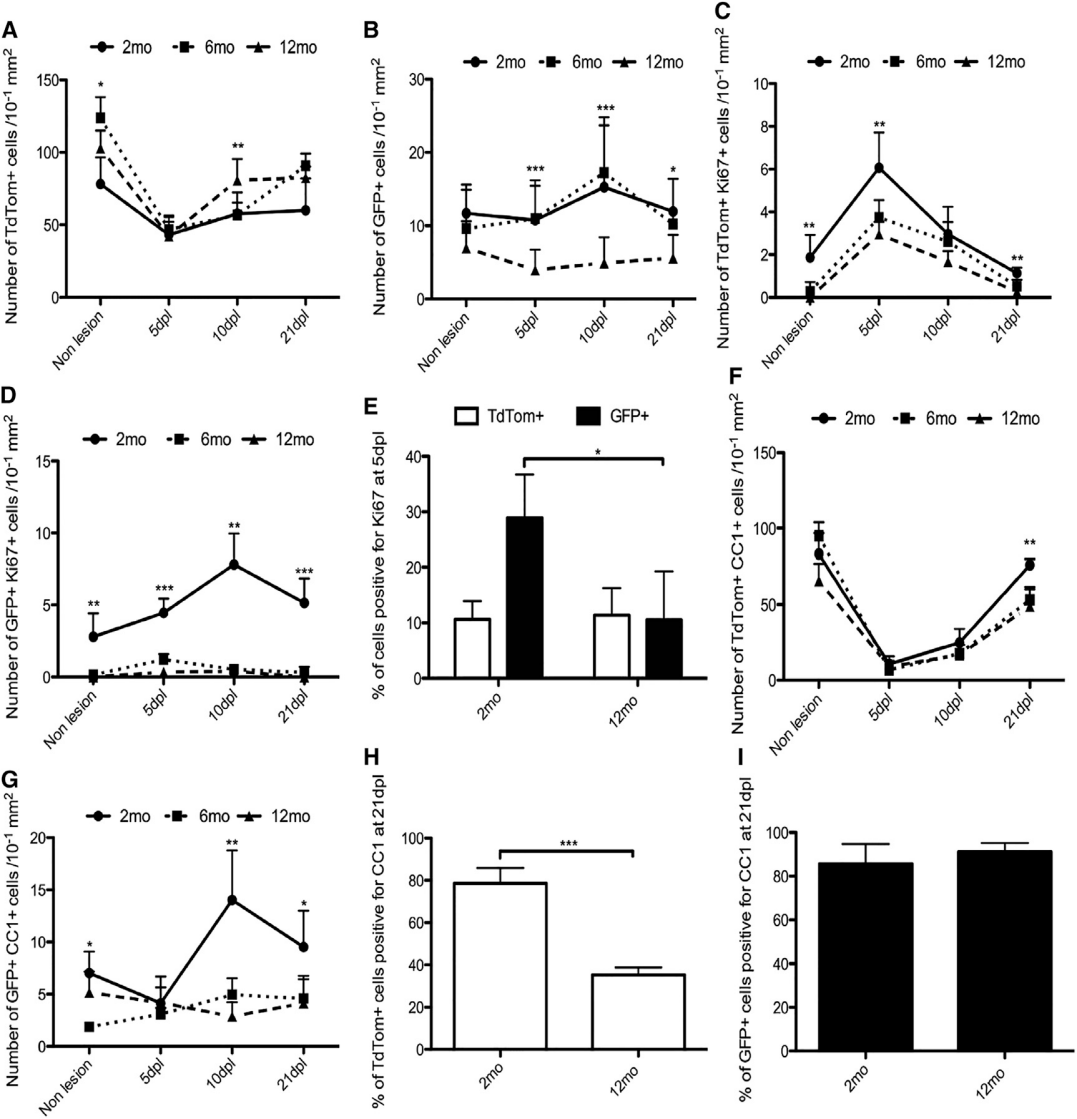
Kinetics of Remyelination in the Aging Corpus Callosum (A) The number of TdTom+ cells in NL tissue is greater in older mice (combining data from 6-month-old and 13-month-old) (p = 0.031 and one-way ANOVA) and at 10 dpl (p = 0.002). (B) The number of GFP+ cells decreases with age (p < 0.001 at 5 dpl and 10 dpl, p = 0.01 at 21 dpl, and one-way ANOVA). (C) The number of TdTom+, Ki67+ cells decreases with age in NL corpus callosum (p = 0.002 and one-way ANOVA) and at 5 dpl (p = 0.004). (D) The number of GFP+/Ki67+ cells decreases with age in the NL corpus callosum (p = 0.001 and one-way ANOVA) and at all times postlesion (5 dpl, p < 0.001; 10 dpl, p = 0.002; and 21 dpl, p < 0.001). (E) The proportion of proliferating GFP+ cells at 5dpl is significantly fewer in 2-month-old animals compared to 12-month-old animals, but remains the same for TdTom+ cells. (F) The number of TdTom+/CC1+ dOLs is decreased in older mice at 21 dpl (p = 0.001 and one-way ANOVA). (G) The number of GFP+/CC1+ cells decreases with age (NL, p = 0.015; 10 dpl, p = 0.001; 21 dpl, p = 0.034; and one-way ANOVA). (H and I) The proportion of TdTom+ dOL lineage cells that is CC1+ at 21 dpl is less in old (13-month-old) than in young (2-month-old) mice (p < 0.001 and Student’s t test), whereas (I) the proportion of vOL lineage cells that is CC1+ at 21 dpl is the same for old and young mice. The data are presented as mean ± SEM (n = 3).

To assess the effect of aging on dorsal (cortical) versus ventral forebrain populations of OL lineage cells and their contributions to remyelination in the corpus callosum, focal demyelination was induced in 6-month- (P170–P198, mean P192) and 13-month-old (P390–P398, mean P393) mice. The density of TdTom+ cells increased in the non-lesioned corpus callosum from 782 ± 185 to 1,029 ± 122 cells/mm^2^ between 2 months and 13 months, while the density of GFP+ cells decreased from 117 ± 39 to 70 ± 37 cells/mm^2^ over the same period (Figures 5A and 5B). Following focal demyelination, this trend continued; the density of TdTom+ cells at 10 dpl and 21 dpl increased modestly with age, while the density of GFP+ cells decreased.

In mice of all ages, there was a marked proliferative response of TdTom+ dOPs at 5 dpl, declining to pre-lesion levels by 21 dpl (Figure 5C). The proliferative response of GFP+ vOPs was slower in 2-month-old mice and practically non-existent in the older (6-month-old and 13-month-old) mice (Figure 5D).

The density of TdTom+, CC1+ dOLs was reduced at 5 dpl in mice of all ages and recovered subsequently, although failed to regain their pre-lesion densities by 21 dpl (Figure 5E). In contrast, the density of GFP+, CC1+ vOLs did not increase significantly between 5 dpl and 21 dpl in the older (6-month-old and 13-month-old) mice (Figure 5F). The proportion of the TdTom+ cell population that co-labeled for CC1 at 21 dpl was significantly decreased in 13-month-old mice relative to 2-month-old mice (from 35% ± 4% versus 79% ± 7%, respectively) (Figure 5G), whereas the proportion of GFP+ cells that was CC1+ was similar in young and old mice (Figure 5H). Therefore, the dOPs are efficiently recruited into the lesion area, but suffer a decline in their rate of OL differentiation, while vOPs appear to be recruited less well, but retain their ability to differentiate rapidly.

### Dorsally and Ventrally Derived OL Lineage Cells Have Different Functional Properties In Vitro

To investigate whether the different remyelination behaviors of dOL versus vOL lineage cells are a result of cell-intrinsic differences or an effect of local environmental factors, we studied them in vitro where cells of both origins could be exposed to the same environment. Dorsally and ventrally derived OL lineage cells were isolated from whole brains of neonatal (P4–P6) and adult mice (mean age P232, 8-month-old) by fluorescence-activated cell sorting for TdTom or GFP fluorescence, respectively. The cells were examined after 48 hr in culture; of the neonate derived cultures, 87% ± 3% of TdTom+ cells and 96% ± 4% of GFP+ cells expressed NG2 and 99% ± 1% of TdTom+ cells and 97% ± 4% of GFP+ cells expressed Olig2, confirming the high purity of the cell preparations and demonstrating that the majority of OL lineage cells were OPs, as expected at that age. In the adult cell cultures, 76% ± 6% of TdTom+ cells and 81% ± 4% of GFP+ cells expressed NG2 and 91% ± 3% of TdTom+ cells and 94% ± 5% of GFP+ cells expressed Olig2. There was no significant difference between the percentage of NG2+ cells in the neonate and adult cultures, or between the percentage of NG2+ cells in the TdTom+ and GFP+ populations of both age groups.

**Figure 6:**
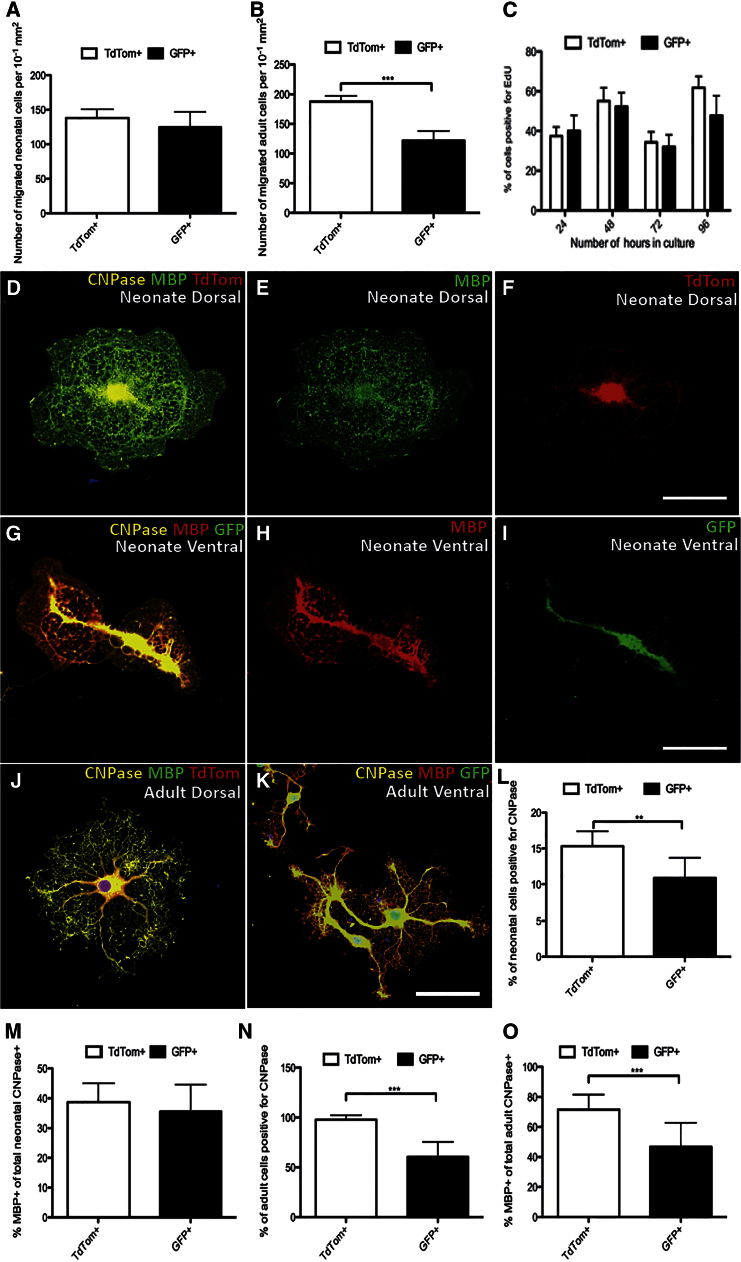
Changes in the Properties of dOL and vOL Lineage Cells In Vitro (A and B) Migratory properties of dOL and vOL lineage cells: no significant difference was detected between the abilities of neonatal TdTom+ dOL versus neonatal GFP+ vOL lineage cells to migrate across a transwell insert within 16 hr (A), whereas significantly more adult (8-month-old) TdTom+ cells migrated compared with adult GFP+ cells (p < 0.001 and Student’s t test) (B). (C) EdU incorporation was the same for TdTom+ and GFP+ neonatal cells. (D–F) Cultured neonatal cells of dorsal origin expressing TdTom, CNPase and MBP (merged) (D), elaborating MBP+ myelin sheets (E), and expressing TdTom (F). (G–I) Cultured neonatal cells of ventral origin expressing GFP, CNPase and MBP (merged) (G), elaborating MBP+ myelin sheets (H), and expressing GFP (I). (J) Adult TdTom+/CNPase+/MBP+ OL with primary and secondary processes, but no myelin sheet formation. (K) Adult GFP+/CNPase+/MBP+ OL with no myelin sheet formation. (L) Significantly more neonatal TdTom+ dOL lineage cells immunolabeled for CNPase, compared with GFP+ vOL lineage cells (p = 0.007 and Student’s t test). (M) Similar fractions of neonatal TdTom+ and GFP+ cells immunolabeled for MBP. (N) Significantly more adult TdTom+ dOL lineage cells differentiated into CNPase+ OLs, compared with GFP+ vOL lineage cells (p < 0.001 and Student’s t test). (O) Significantly more adult TdTom+ cells than GFP+ cells differentiated into MBP+ OLs (p < 0.001 and Student’s t test). The data are presented as mean ± SEM (n = 3).

To assess cellular migration, a transwell assay was performed. In neonatal brain-derived cultures, there were no significant differences in the numbers of TdTom+ or GFP+ cells that had migrated through the membrane in the 16 hr of the experiment (Figure 6A). However, in the 8-month-old brain-derived cultures, significantly more TdTom+ cells migrated through the membrane compared with GFP+ cells (Figure 6B). These data suggest that adult brain derived dOPs migrate more readily in culture, compared to their ventral counterparts.

To assess proliferative potential, neonatal cells were cultured for 24, 48, 72, or 96 hr, before incubating for 6 hr in EdU. Both TdTom+ and GFP+ cell populations incorporated EdU to approximately the same extent (34%–62% of TdTom+ cells versus 32%–52% of GFP+ cells) (Figure 6C). Cells from 8-month-old brains were assessed after 96 hr in culture, but very limited proliferation was detected; 0.9% ± 0.7% of TdTom+ cells stained positive for EdU and 1.1% ± 0.8% of GFP+ cells (p = 0.423; Student’s t test). Therefore, no inherent difference in proliferative potential was detected between the TdTom+ and GFP+ cell populations.

To assess differentiation efficiency and fate, the cells were cultured for 8 days, fixed, and stained for 2′,3′-cyclic-nucleotide 3′-phosphodiesterase (CNPase), a myelin-associated enzyme expressed at the onset of OL differentiation, and myelin basic protein (MBP), a major protein component of the myelin sheath. Both TdTom+ dOPs and GFP+ vOPs differentiated into CNPase+, MBP+ cells with the classic branching morphology of OLs. Neonatal cells typically generated myelin sheets (Figures 6D–6I), which were not observed in the 8-month-old cell cultures (Figures 6J and 6K). In both neonatal and 8-month-old cell cultures, a significantly greater proportion of TdTom+ cells differentiated into CNPase+ OLs, compared with their GFP+ counterparts (Figures 6L and 6N). Of the neonatal OLs that expressed CNPase, similar numbers of TdTom+ and GFP+ cells co-expressed MBP. However, in the 8-month-old cultures, a significantly greater fraction of TdTom+, CNPase+ cells than GFP+, CNPase+ cells co-expressed MBP (Figure 6H). These data suggest that dOPs differentiate more readily into MBP+ dOLs in culture, compared to their ventral counterparts.

### Genetic Ablation of Dorsally Derived OL Lineage Cells Causes a Reduction in Remyelination Efficiency

**Figure 7:**
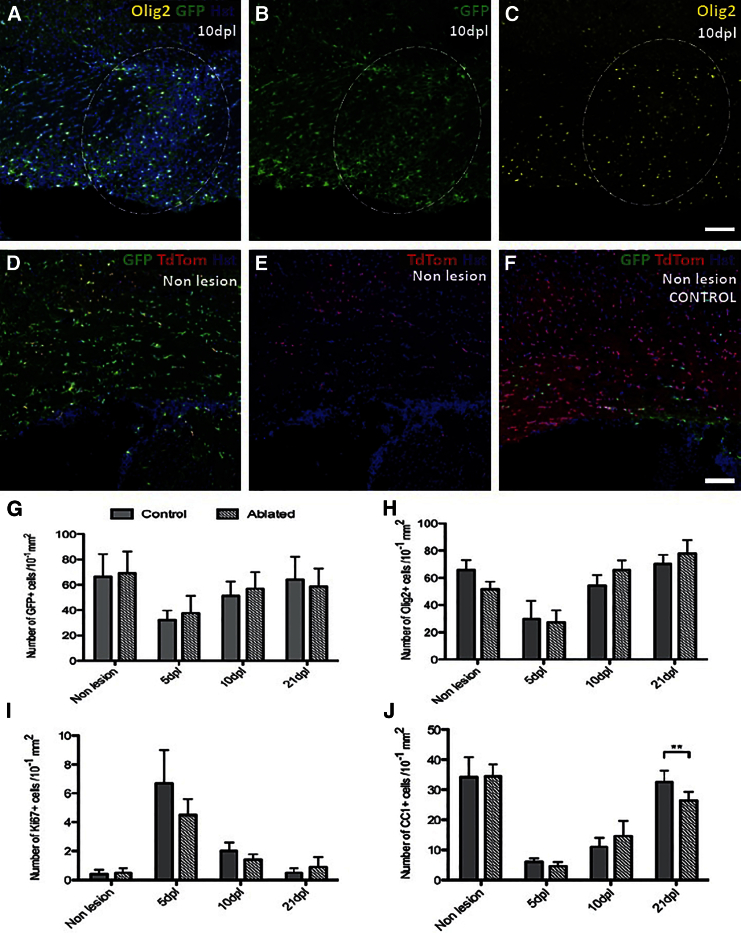
Genetic Ablation of Dorsally Derived, Emx1+ Cells Leads to a Reduction in the Number of CC1+ Cells at 21 dpl (A–C) Ventral lesioned white matter showing expression of Olig2, GFP and the nuclear marker Hoechst dye (Hst) (A), GFP alone (B), and Olig2 alone (C). The percentage of Olig2+ cells (C) co-expressing GFP (B) (merged in A) was found to be 95.9% ± 2.7%, confirming efficient transgene expression in *Emx1-Cre/Sox10-GFP-DTA* corpus callosum at 10 dpl (lesion demarcated by the white dashed line). (D–F) Ventral non-lesioned white matter showing expression of GFP, TdTom, and Hst (D) and TdTom alone (E) in triple transgenic *Emx1-Cre/Sox10-DTA/Sox10-GFP-TdTom* mice, and expression of GFP, TdTom, and Hst in control, non-ablated white matter (F), revealing that the DTA ablation strategy deleted the majority of Emx1+ cells from the CC. The scale bar represents 100 μm. (G) There was no significant difference between the number of GFP+ cells in ablated and control mice at any of the time points studied. (H) No significant difference was detected in the number of Olig2+ GFP+ cells at any time point. (I) No significant difference was detected in the number of Ki67+ GFP+ cells at any time point. (J) There was a significant reduction in the CC1+ cell number at 21 dpl in the ablated mice compared with controls (p = 0.007 and Student’s t test). The data are presented as mean ± SEM (n = 3). The scale bars represent 100 μm.

A genetic ablation strategy was used in which *Emx1*-positive dOPs and dOLs were eliminated at source by the targeted expression of diphtheria toxin A fragment (DTA). 2-month-old (P61–P74, mean age P67) mice carrying the *Em1-Cre* and *Sox10-GFP-DTA* transgenes were used, in which GFP is excised along with a poly(A) signal following Cre recombination, thus activating DTA and killing the cell (Kessaris et al., 2006)(Figures 7A–7C). To determine the efficiency of Cre recombination and the associated deletion of Emx1-positive cells, the *Emx1-Cre* line was crossed to both the *Sox10-DTA* and the *Sox10-GFP-TdTomato* lines. In the triple transgenic offspring, *Emx1* expression by dorsally derived cells should result in recombination and expression of DTA. Any cells escaping DTA expression would be expected to label with TdTomato and hence provide an indication of the success of this genetic deletion strategy (Figures 7D–7F). 8.9 ± 3.5 TdTomato-positive cells per 10^−1^ mm^2^ were present in the triple transgenic mice, compared to 78.2 ± 18.5 cells per 10^−1^ mm^2^ in control mice (*Emx1-Cre/Sox10-GFP-TdTomato*). This would suggest that 85 ± 5.5% of the Emx1-positive population was destroyed at source with the inducible DTA genetic deletion strategy.

Lysolecithin injection into the corpus callosum was performed to create a focal area of demyelination, and the subsequent remyelination process was compared between *Emx1-Cre/Sox10-DTA* ablated mice and control animals (*Sox10-DTA*). No significant difference was detected in the total GFP+ cell number between ablated and control animals at any time point (Figure 7G). Nor did we detect a difference in the number of Olig2+, GFP+ cells (Figure 7H), nor in the number of Ki67+, GFP+ cells (Figure 7I). However, there were significantly fewer CC1+ cells in ablated animals compared to controls at 21 dpl (32.5 ± 3 cells/mm^2^ in control animals, compared with 26.4 ± 2 cells/mm^2^ in ablated animals; p = 0.007 and Student’s t test) (Figure 7J). This suggests an impairment of the differentiation phase of remyelination in the ablated mice.

## Discussion

OPs emerge from discrete parts of the ventral and dorsal VZ in the embryonic spinal cord and forebrain (Cai et al., 2005, Fogarty et al., 2005, Vallstedt et al., 2005, Kessaris et al., 2006; reviewed by Richardson et al., 2006). Previous studies have identified similar electrophysiological properties of the ventrally and dorsally derived populations of both spinal cord and brain (Clarke et al., 2012, Tripathi et al., 2011). Moreover, when ventrally or dorsally derived OL lineage cells in the developing forebrain were ablated by targeted DTA expression, the remaining population(s) proliferated and migrated more than normal to compensate for the loss (Kessaris et al., 2006). These observations suggested that OL lineage cells derived from different parts of the VZ are functionally equivalent and can substitute for one another during development. However, the possibility remained that there might be functional differences that only become apparent during pathology. In support of the latter idea, Zhu et al. (2011) demonstrated that, in response to a focal demyelination injury in the ventral spinal cord, an unexpectedly large number of newly differentiated OLs were dorsally derived, suggesting that dOL lineage cells might be particularly adapted for remyelination. To investigate this possibility further, we made use of a dual-reporter mouse line in which OL lineage cells can be differentially marked according to their sites of origin in the ventral or dorsal VZ, allowing side-by-side comparison of their differentiation and remyelination capacities in vivo and in vitro.

In the normal adult spinal cord, the ventral funiculus is dominated by pMN-derived OL lineage cells with infrequent cells from the dorsal VZ. However, in response to focal demyelination in the ventral funiculus, the rare dOPs undergo a marked proliferative response, with prompt recruitment into the lesion core and differentiation into mature OLs, so that by 60 dpl, dorsally derived cells outnumber their ventrally derived counterparts and are the major player in the oligodendrocyte remyelination response. This also applies to the component of spinal cord demyelination that is mediated by Schwann cells where dorsally derived cells are the dominant contributors. It is uncertain whether this reflects an inherent propensity for these cells to differentiate into Schwann cells compared with ventrally derived cells or whether this is simply a function of their greater proliferative response resulting in greater numbers of dorsally derived cells residing within the lesion niches where Schwann cell remyelination occurs (Zawadzka et al., 2010).

As in the spinal cord, ventrally and dorsally derived OP populations sequentially populate the developing forebrain, raising the question of whether similar differences might be detected in their relative responses to demyelination in the adult forebrain. The corpus callosum and adjacent subcortical white matter is populated mainly by cortex-derived dorsal OL lineage cells with an ∼20% contribution of OL lineage cells from the ventral forebrain (Tripathi et al., 2011). When we induced focal demyelination in the corpus callosum, the remyelination response was dominated by the dorsal (cortex-derived) population. A marked proliferative response of dOPs was detected at 5 dpl, with differentiation into CC1+ dOLs and restoration of pre-lesion numbers of dOL lineage cells complete by 21 dpl. On the other hand, while numerous Ki67+ vOPs were detected in the lesion at 5 dpl and 10 dpl, very few of these seemed to differentiate into CC1+ vOLs, presumably persisting as vOPs in the longer term. Thus, in the corpus callosum, both dOPs and vOPs react differently to demyelination, with dOPs demonstrating a strong regenerative propensity. However, due to their predominance in this tract, it is not possible to determine whether dorsally derived progenitors made a disproportionately high contribution to remyelination compared with their ventral counterparts, as is the case in the ventral spinal cord.

The rate of remyelination is strongly reduced in aged animals. We asked whether this age-related decline is a function of either the dorsal or ventral population of OL lineage cells, or both. We created demyelinated lesions in the spinal cords (ventral funiculus) of older mice (6-months-old or 13-months-old) and assessed the response of the dorsally and ventrally derived OL populations. Comparing remyelination of lesions in older versus younger mice, we found that dOPs were more efficiently recruited in older animals, but their ability to differentiate into CC1+ OLs was reduced. In contrast, vOPs were less efficiently recruited into lesions in the older mice but, once recruited, their ability to differentiate was unhindered. Thus, the two populations show very different age-associated functional changes. However, the final density of differentiated OLs within remyelinated spinal cord lesions was the same in older versus younger mice, suggesting that increased recruitment of dOPs compensates for their reduced differentiation capacity. Shen et al. (2008) have demonstrated an age-associated decrease in histone deacetylase (HDAC) recruitment, leading to prolonged expression of differentiation inhibitors, and ultimately an impairment of OP differentiation. Perhaps HDAC recruitment failure is more pronounced in the dOP population and results in increased susceptibility to age-associated differentiation block. Despite an apparent age-associated slowing in the rate of differentiation, it remains a possibility that the proportion of dorsally derived OLs increases over the more prolonged course of remyelination in aged animals.

In the aging corpus callosum, dOPs continued to dominate the remyelination process, remaining the more numerous population both within the lesion and in neighboring unlesioned tissue. We detected an age-associated decline in recruitment of dOPs into the lesion, as well as a decline in the number of Ki67+ proliferating dOPs and of CC1+ dOLs. In line with the reduced proliferation index, the proportion of dOPs that had differentiated by 21 dpl also decreased with aging. Thus, as for the spinal cord, there is an age-associated decline in the proliferation/differentiation activity of the dOP population. This is consistent with the overall age-related decline in OP proliferation rates—coupled to declining rates of production of differentiated OLs—in the normal healthy mouse (Psachoulia et al., 2009, Lasiene et al., 2009, Young et al., 2013).

vOPs decreased in total number within the non-lesioned and lesioned corpus callosum, demonstrating a corresponding reduction in their proliferation response to focal demyelination. However, the aged vOPs appeared to be able to maintain a high efficiency of differentiation into CC1+ OLs by 21 dpl. Therefore, vOPs in the aging corpus callosum, as in the aging spinal cord, show a sustained differentiation capacity with advancing age.

The different settling patterns of dorsal and ventral OPs and OLs within the adult CNS raised the possibility that their functional heterogeneity might be the result of extrinsic environmental factors, rather than intrinsic properties of the cells themselves. To investigate this possibility, the two populations were FACS-purified and cultured under identical conditions. In both neonatal and adult brain-derived cultures, we found differences in the in vitro properties of the ventrally and dorsally derived cells. Adult brain dOPs showed reduced viability and very little proliferation, so limiting the total cell numbers obtained and hence the number of factors and time points that could reasonably be tested. Nevertheless, adult dOPs showed enhanced migration, an increased tendency to differentiate toward an OL fate, and a greater efficiency of differentiation into MBP-positive OLs compared to their ventral counterparts. Therefore, in vitro analysis confirmed that dorsal and ventral OPs differ intrinsically; the two populations show different migration and differentiation capacities when in an identical environment. This heterogeneity appeared to become more apparent in the adult brain, compared with the neonatal brain, and may reflect a differential susceptibility of the two populations to aging changes, as identified in vivo.

No differences were detected in the proliferation capacities of the two populations, conflicting with the in vivo finding of a greater proliferative response in dOPs following focal demyelination. The microenvironment created by demyelination is not a feature of the cell culture and so this discrepancy may reflect the fact that both populations proliferate to a similar extent in culture medium, but dOPs show enhanced responsiveness to a demyelinating insult, mediated via enhanced proliferation initially.

We cannot exclude the possibility that the apparent efficiency of differentiation of the dOPs may be a function of the greater number of proliferating precursors from the dorsally derived pool. However, our in vitro studies reveal that in an identical environment, with an equivalent proportion of Ki67+ proliferating cells, the dOPS demonstrate a greater efficiency of differentiation into MBP-positive OLs compared to their ventral counterparts. If these properties persist in vivo, the dOP contribution to remyelination is likely to be a result of increased efficiency of both recruitment and differentiation. Such synergism could feasibly prime the cells for an optimal response to a demyelinating insult.

The rapid proliferative response of dOPs following focal demyelination shares features characterized by transit amplifying cells of the hematopoietic system. Transit amplifying cells are partially lineage-committed and are able to respond rapidly to injury with a prompt proliferative response and rapid regeneration of the compromised cell type. Our in vitro analysis (this study) revealed a greater tendency for dOPs to generate OLs compared with their ventral counterparts. Further studies to characterize the molecular markers and gene expression profiles of the two OP populations might shed further light on their phenotypic differences and the reasons behind them.

A genetic ablation strategy was applied to the forebrain and 85 ± 5.5% of Emx1+ dorsally derived cells were destroyed at source. The proliferation and recruitment phases of remyelination appeared to be unaffected, suggesting that either the ventrally derived population or the surviving 15 ± 5.5% of dorsally derived cells were able to compensate in the early stages of remyelination. However, there was a modest reduction in the number of CC1+ OLs within the lesion at 21 dpl in ablated animals compared with control. Therefore, ablation of ∼85% of dOPs is sufficient to interfere with the differentiation phase of remyelination, suggesting a failure of compensation by the vOPs. Future studies to address the extent of this failure of compensation, particularly in the context of aging, may provide further insight into the functional heterogeneity of the dorsal and ventral populations.

In conclusion, the developmental heterogeneity of OPs determines their phenotypic properties in the adult CNS. The cells’ response to demyelination, contribution to remyelination, and susceptibility to age-associated functional decline is influenced by their developmental origin. Analysis of the gene expression profiles of the dorsal and ventral OPs, in both the diseased and aging environments, could reveal potential targets for therapeutic enhancement of remyelination, particularly with regard to overcoming the age-associated decline in the efficiency of differentiation of OPs into mature, myelinating OLs.

## Experimental Procedures

### Transgenic Mice

*Sox10-loxP-eGFP-polyA-loxP-TdTomato* reporter mice (*Sox10-GFP-TdTom*) express enhanced GFP in all OL lineage cells (OPs and OLs) in the absence of Cre activity. They were crossed to either *Msx3-Cre* (Fogarty et al., 2005) or *Emx1-Cre* (Kessaris et al., 2006) lines to induce TdTom expression in OPs developing from the dorsal spinal cord VZ or the cortical VZ, respectively. To study the effect of genetically ablating the dorsally derived, Emx1-positive cells of the forebrain, the *Emx1-Cre* line was crossed to the *Sox10-loxP-GFP-poly(A)-loxP-DTA* mouse (Kessaris et al., 2006).

Mice were kept on a 12 hr dark/light cycle and the day of birth designated P0. All animal experiments conformed to the UK Animals (Scientific Procedures) Act 1986 and were approved by the UCL or Cambridge University local ethical committees before licensing by the UK Home Office.

### Spinal Cord Demyelination

A focal area of primary demyelination was created in the caudal thoracic ventral funiculus of the spinal cord by injection of 1% (v/v) lysolecithin (Woodruff et al., 2004, Zawadzka et al., 2010). Animals of equivalent age that had not undergone surgery provided non-lesioned controls.

### Corpus Callosum Demyelination

General anesthesia and perioperative analgesia were provided as for the spinal cord demyelination model. Using a Stoetling stereotactic frame, the dorsal aspect of the skull was clipped and surgically prepared. A midline incision was made from just caudal to the eyes to the occipital crest. A small hole was drilled in the skull at 0.08 mm lateral and 0.05 mm rostral to Bregma. Unilateral injection of 2 μl of 1% (v/v) lysolecithin was delivered at a depth of 0.24 mm below the brain surface, into the corpus callosum, using a 5 μl Hamilton syringe. The needle was left in position for 3 min to allow toxin dispersal. Mice were placed in a warm incubator and monitored for full recovery.

### In Vivo EdU Assay

Following lysolecithin injection, mice received EdU (5-ethynyl-2′-deoxyuridine, Invitrogen) by intraperitoneal injection (50 μg/gram of body weight/day) on 2 consecutive days. Treated mice were sacrificed 24 hr after the second EdU dose. Tissue sections were stained using the “Click-iT Plus” EdU Alexa Fluor 647 Imaging Kit (Invitrogen).

### Tissue Processing

Mice were terminally anaesthetized and fixed by intracardiac perfusion at 5, 10, 21, or 60 dpl induction using 4% (w/v) paraformaldehyde (PFA) in 0.1 M PBS. Spinal cords or brains were removed, postfixed in 4% PFA for 2 hr at 20°C–25°C, cryoprotected with 20% (w/v) sucrose for 24–48 hr, embedded and frozen in OCT medium, and stored at −80°C. Tissues were sectioned at 12 μm and collected onto poly-L-lysine-coated glass slides.

### Immunocytochemistry and Microscopy

Spinal cord sections were pre-treated with blocking solution (8% [v/v] normal goat serum and 0.1% [v/v] Triton X-100 in PBS], incubated with primary antibodies overnight at 4°C, and then secondary antibodies for 2 hr at 20°C–25°C. The following primary antibodies were used at 1:1,000 dilution unless stated otherwise: GFP (chicken, Aves lab), Olig2 (rabbit, Millipore), CC-1 (anti-adenomatous polyposis coli, APC) (mouse, 1:100, Calbiochem), Nkx2.2 (mouse, 1:200, DSHB), NG2 (rabbit, 1:200, Millipore), Ki67 (rabbit, Abcam), Periaxin (rabbit, 1:3,000, from Peter Brophy, Centre for Neuroscience Research, University of Edinburgh, UK), SCIP/Oct-6 (rabbit, 1:4,000, from Dr. John Bermingham Jr., McLaughlin Research Institute, Montana, USA), MBP (rat, 1:350, Serotec). Secondary antibodies were: Alexa Fluor 488-, 568-, or 647-conjugated goat antibodies against mouse, rat, rabbit, chicken, or goat IgG or IgG2b (1:1,000, all from Invitrogen). Cell nuclei were visualized by poststaining with Hoechst 33258 (Sigma, 1:1,000). For cell counts, at least three sections per animal from three to six mice were examined using a Leica SP5 confocal microscope. Data are quoted as mean ± SEM.

Statistical analysis was performed using the Statistical Package for the Social Sciences software. The data were analyzed for normal distribution and interactions. A two-tailed Student’s t test was performed to assess the statistical significance of the variation in single time point cell counts. The cell type and the time point were found to interact and so were analyzed separately. To determine the significance of changes in the cell types over multiple time points, one-way and two-way ANOVAs were performed, with Bonferroni and Tamhane post hoc tests as appropriate based on the presence of homogeneity of variance. Where data were not normally distributed, a Mann-Whitney U-test or Kruskal-Wallis test was performed. Significance levels were set at 5% and the data are presented graphically as: ^∗^(p < 0.05), ^∗∗^(p < 0.01), and ^∗∗∗^(p < 0.001).

### OP Isolation and Culture

Brains from adult mice (6- to 9-months-old) were placed into cold Neurobasal medium (NB) (Gibco) with 1% (v/v) penicillin streptomycin (Sigma), cut into small fragments, and transferred to a digestion mix consisting of 4 ml of NB, 160 μl papain (Worthington), 40 μl DNase 1 Type IV (Sigma), and 40 μl of L-cysteine (Sigma). The tissue was incubated at 37°C for 1 hr in the digestion solution, followed by mechanical dissociation using serum-coated, fire polished glass pipettes. The papain was inactivated by trituration in 3 ml of NB containing 500 μg/ml of BSA Fraction V (i.e., 25 mg) (Sigma), 40 μg/ml of DNase I Type IV, and 1 mg/ml of trypsin inhibitor (Sigma) and diluted in 20 ml NB before centrifugation for 8 min at 250 × *g*. The pellet was resuspended in 15 ml NB and passed through a 70 μm pore-diameter nylon filter to achieve a single cell suspension. The cell suspension was resuspended in a 30% (w/v) Percoll solution (GE Healthcare) and centrifuged at 800 × *g* for 20 min. The top myelin fraction was aspirated and discarded, allowing collection of the OL-rich layer, which was diluted in 100 ml NB and centrifuged at 250 × *g* for 8 min. The cell pellet was resuspended in neuronal media for optogenetics (NEMO, Cell Guidance Systems UK) and supplements for optogenetic survival (SOS, Cell Guidance Systems UK), supplemented with 1% (w/v) DNase, 5% (w/v) BSA (Sigma), 20 ng/ml basic fibroblast growth factor (bFGF/ Fgf-2; Peprotech), and 20 ng/ml platelet derived growth factor (Pdgf-AA; Peprotech). FACS was used to isolate the GFP-positive and TdTom-positive populations using a three laser MoFlo cytometer and cells were collected into NEMO-SOS containing 1% (v/v) penicillin streptomycin and PDGF/FGF (each at 20 ng/ml). Cells were seeded at 50,000 per 12 mm plastic coverslip (Thermoscientific). Wells and coverslips had been pre-coated with 0.05% (w/v) polyornithine (Sigma) for 3 hr at 37°C, rinsed with sterile distilled water, and incubated with 5 μg/ml laminin (Sigma) diluted in Hank’s buffered salt solution (HBSS, GIBCO) for 3 hr at 37°C.

Cells were maintained in 8.5% (v/v) CO_2_ at 37°C. Growth factors were supplemented daily for 2 days and the medium then switched to NB media containing 1% (v/v) GlutaMAX (Gibco,), 2% (v/v) B27 (Gibco), 50 μg/ml apotransferrin (Sigma), 0.2% (w/v) bovine pancreatic insulin (Sigma), 1% (v/v) penicillin streptomycin, 0.1% (w/v) N-acetylcysteine (Sigma), and PDGF/FGF (concentrations as above). To study cell proliferation, PDGF and FGF were added daily and the medium changed every other day. Cells were then incubated for 6 hr in 10 μM EdU and fixed in 4% (w/v) PFA at 20°C–25°C for 10 min and rinsed in PBS. To study cell differentiation, the growth factors were withdrawn after 48 hr and the medium changed every other day for a further 6 days before fixation. The immunofluorescence labeling protocol was used as described above for tissue sections, with the additional use of the following primary antibodies: GFAP (rabbit, 1:500, Dako) and CNPase (mouse, 1:200, AbCam).

Cell migration was assessed by seeding 10,000 cells onto an 8 μm pore size, PET cell culture insert (Millipore). The insert was placed into a well containing NEMO-SOS plus PDGF/FGF (each at 30 ng/ml) and incubated in 8.5% (v/v) CO_2_ at 37°C for 16 hr before fixation. Cell nuclei were visualized by poststaining with Hoechst 33258. Cells that had successfully migrated were imaged and quantified.

Neonatal OPs were isolated, cultured, and analyzed as for the adult cells, except that the Percoll gradient centrifugation was replaced by a wash in 1 ml of 4% (w/v) BSA in NB medium.

## Author Contributions

A.H.C. conducted the experiments, with assistance from R.B.T. and R.J.M.F. All authors contributed to the design of the experiments. A.H.C. and R.J.M.F. wrote the paper, with assistance from R.B.T. and W.D.R.
